# Daily Step Count 2 Years After Anterior Cruciate Ligament Reconstruction and Associations With Cartilage Health and Knee Symptoms and Function

**DOI:** 10.1177/19417381251384327

**Published:** 2025-10-22

**Authors:** Britt Elin Øiestad, Ashley A. Williams, Matthew R. Titchenal, Amelie M. Lutz, Constance R. Chu

**Affiliations:** † Department of Rehabilitation Science and Health Technology, Oslo Metropolitan University, Oslo, Norway; ‡ Department of Orthopedic Surgery, Stanford University, Stanford CA and Veterans Affairs Palo Alto Health Care System, Palo Alto, CA; § Department of Mechanical Engineering, Stanford University, Stanford, CA; InSciTech, Inc., Mountain View, CA; ‖ Department of Radiology, Kantonal Hospital Muensterlingen, Switzerland; ¶ Department of Orthopedic Surgery, Stanford University, Stanford, CA

**Keywords:** activity level, cartilage morphology, knee pain, knee-related quality of life, symptoms

## Abstract

**Background:**

People suffering anterior cruciate ligament (ACL) injuries are at increased risk for development of osteoarthritis (OA). This study investigated associations between daily step count, cartilage degeneration and patient-reported outcomes 2 years after ACL reconstruction (ACLR).

**Hypothesis:**

Daily step count is associated with cartilage health and patient-reported knee symptoms and function 2 years after ACLR.

**Study Design:**

Cross-sectional.

**Level of Evidence:**

Level 4.

**Methods:**

We analyzed data from 34 patients (18 female), aged 33.4 ± 10.8 years with stable knees recruited from the community 2 years after primary ACLR. Mean daily step count was measured using an activity tracker (FitBit) over a 7-day collection period. Cartilage morphology on magnetic resonance imaging (MRI) was graded across multiple joint areas. Knee symptoms and function were assessed by the Knee injury and Osteoarthritis Outcome Score (KOOS) subscales pain, symptoms, activity of daily living (ADL), sport/recreation (sport/rec), and knee-related quality of life (QoL) using published thresholds for patient acceptable symptom state (PASS). Analyses were adjusted for age, sex, and body mass index.

**Results:**

The mean (SD) daily step count was 9276 (3199). At least 1 cartilage abnormality was present on morphological MRI in 20% of ACLR knees. The mean (SD) KOOS values were: pain 94 (7), symptoms 92 (8), ADL 91 (10), function in sport/rec 85 (14), and knee-related QoL 56 (22). Failure to achieve PASS rates were 76% for ADL; 59% for QoL, 18% for pain, 35% for sport/rec, and 0% for symptoms.

**Conclusion:**

Daily step count was not associated with cartilage health or knee symptoms and function 2 years after ACLR. However, a high proportion of participants with reported unacceptable ADL and QoL 2 years after ACLR.

**Clinical Relevance:**

The proportion with unacceptable PASS for ADL and QoL in participants with stable knees after ACLR indicates a need to optimize rehabilitation and improve post-ACLR recovery.

People with anterior cruciate ligament (ACL) injury are at high risk of developing post-traumatic radiographic and symptomatic knee osteoarthritis (OA).^14,17,32^ The initial trauma to the joint structures, particularly in the meniscus, cartilage, and subchondral bone, combined with altered biomechanics, persistent subclinical inflammation, and neuromuscular deficits, comprise leading theories behind the accelerated development of degenerative changes.^4^ Additional injuries to meniscal and other joint structures further increase the risk for development of post-traumatic knee OA in patients with ACL injury.^32^

Physical activity and exercise are recommended for patients with knee injuries to improve knee function and to assist in mitigating known risk factors for knee OA such as excessive body weight and muscle weakness.^32^ In addition, physical activity influences the joint positively because the mechanical loading stimulates the extracellular matrix synthesis and suppresses proinflammatory driven catabolism.^15,26^ A systematic review found that knee joint loading exercises did not seem to adversely affect molecular biomarkers related to cartilage health and inflammation in people at risk for or with established OA.^3^ However, there is still conflicting evidence regarding the influence of physical activity on joint degeneration and OA.^15^ Due to the large variation in OA disease, more precise characterizations of structural disease state and physical activity than radiographs and patient-reported outcomes are needed to improve understanding of the effects of activity on cartilage health. Conventional and advanced quantitative magnetic resonance imaging (MRI) improve the ability to detect early cartilage changes present in the initial stages of OA. For instance, compositional MRI such as quantitative MRI ultrashort echo time (UTE) T2* (UTE-T2*) has been shown to be sensitive to deep tissue matrix changes after ACL reconstruction (ACLR) when cartilage still appears normal by arthroscopy and conventional MRI.^6,29,34^ Thus, MRI assessments of cartilage health are considered more sensitive than radiographs to early OA disease states.^5^

Self-reported questionnaires are often used to measure physical activity levels, but they are subjective and do not typically include all knee-loading activities. Further, self-reports tend to overestimate physical activity level.^27^ Objectively measured daily step count is one method to better capture the overall knee-loading physical activity. Bell et al^1^ compared physical activity levels and daily step counts by accelerometer between patients 28 months after ACLR and healthy controls. They found that the group with ACLR had significantly less time of moderate-to-vigorous physical activity and fewer daily steps compared with the healthy control group, but they detected no association between daily step count and knee function. A separate study of people with ACLR also found no association between daily steps and knee-related quality of life (QoL).^9^

Few studies have investigated whether objectively measured physical activity level correlates with early cartilage changes detected on MRI or function in patients early after ACLR who are at risk for OA but do not yet have clinical disease.^7-9,35^ More studies are needed to better understand the relationships between step counts, cartilage health and knee symptoms and function after ACLR. The objective of this study was to investigate the associations between daily step count with cartilage morphology and cartilage subsurface matrix and the association between daily step count and patient-reported knee symptoms and function 2 years after ACLR.

## Methods

The study had a cross-sectional design, recruiting participants who had ACLR 2 years previously from the community, which therefore included multiple surgeons and institutions. Participants responded to Institutional Review Board (IRB) approved advertisements and emails. Patients who had primary ACLR in either limb (no revision ACLR), were aged between 18 and 60 years, body mass index (BMI) <30 kg/m^2^, no injections to knees or other joints in the preceding 6 months, self-reported knee stability, and an intact ACL graft (on morphological MRI and side-to-side difference of <5 mm measured by KT-1000 arthrometer) were included. The reason for including participants with stable knees of <5 mm side-to-side difference by KT-1000 was to avoid the confounding effects of failed ACLR. A total of 50 people (23 females and 27 males) were examined in the period 2014 to 2017 at a mean of 2.2 ± 0.2 years after ACLR.^29^ All study participants signed an IRB-approved informed written consent at the time of inclusion.

### Assessment of Daily Step Count

Daily step count was measured using a FitBit Charge HR (Fitbit Inc). Participants wore the tracker on their nondominant wrist for 7 consecutive days, starting the morning after the assessment day, and they were told to complete a written activity log. After the 7-day period, participants were asked in the activity log whether the 7-day period represented their typical physical activity. Those who reported abnormal activity for varying reasons (eg, sickness, injury, etc) were excluded from the analysis. Total number of steps was recorded for each day in the 7-day collection period. The average step count of 4 consecutive days was calculated in this study, preferably for days 2 to 5 if that was available, otherwise days 3 to 6 to avoid observation bias.

### Assessment of Cartilage Morphology

The study participants underwent MRI examination of the injured knee on a 3 T scanner (MR 750 GE Healthcare) with transmit-receive 8-channel knee coil (InVivo Inc) as described by Williams et al.^33^ Cartilage morphology was assessed from proton density fast spin echo sequences with fat saturation (PD FSE FS) acquired in 3 orthogonal planes, a nonfat saturated sagittal PD FSE sequence and a three-dimensional (3-D) FSE (CUBE) sequence. Morphologic grading was performed by an experienced radiologist in 7 tibiofemoral cartilage subregions: in central and posterior weightbearing zones of the medial femoral condyle (MFC) and lateral femoral condyle (LFC) and the central medial tibial plateau (MTP) and lateral tibial plateau (LTP) and the posterior LTP. The cartilage was graded using a modified 5-point Outerbridge system as described by Potter et al^22^: Grade 0 indicated intact cartilage, Grade 1, chondral softening or blistering with an intact surface; Grade 2, shallow superficial ulceration, fibrillation, or fissuring involving less than 50% of the depth of the articular surface; Grade 3, deep ulceration, fibrillation, fissuring, or a chondral flap involving 50% or more of the depth of the articular cartilage without exposure of subchondral bone; and grade 4, full-thickness chondral wear with exposure of subchondral bone. Grade 0 was compared with Grades 1 to 4.

### Assessment of Subsurface Cartilage Composition

**Figure 1.**
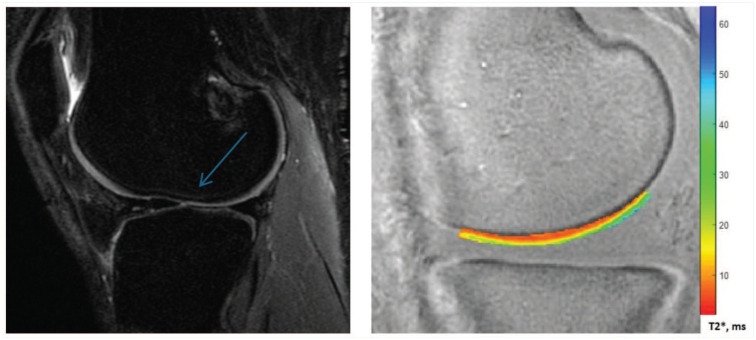
Left, Image of sagittal fat-sat PD. Blue arrow shows a Grade 2 abnormality just at the end of the ROI for central lateral femur condyle. Right, ROI for UTE-T2* scoring of the same image. PD, proton density; ROI, region of interest; UTE-T2*, ultrashort echo time T2*.

Cartilage subsurface matrix organization was assessed with quantitative MRI (qMRI) using a radial-out, 3-D Cones acquisition. A series of T2*-weighted MRI scans were acquired at 8 nonuniformly spaced echo times (TEs) ranging from 32 microseconds to 16 milliseconds, 12 cm field of view, 3 mm slice thickness, and 384 in plane matrix interpolated to 512 for an effective resolution of 234 µm × 234 µm. UTE-T2* maps were generated from the 3-D Cones images with a mono-exponential pixel-by-pixel T2-fit routine using commercial software (Olea Medical). We assessed cartilage composition in focal tibiofemoral strips of deep cartilage, which we term “tread mark” regions of interest (ROI) and which are largely consistent with known regions of cartilage contact during common daily activities,^28^ because these subregions have previously demonstrated compositional changes in patients with ACLR.^34^ Analyzing deep weightbearing zones gives the opportunity to visualize joint tissue damage before the development of irreversible cartilage changes. Mean UTE-T2* values for each ROI were recorded for analysis. Figure 1 shows example images from a sagittal PD scan used in Outerbridge grading and a UTE-T2* map for a specific ROI.

### Assessment of Symptoms and Function

Patient-reported knee symptoms and function was assessed by the Knee injury and Osteoarthritis Outcome Score (KOOS).^25^ KOOS has 5 subscales; pain, other symptoms, activities of daily living (ADL), sport and recreation (Sport/Rec), and knee-related QoL, all of which sum up to a number between 0 and 100, where 0 is the worst possible score, and a value of 100 indicates normal knee function. The questionnaire is validated for people with post-traumatic knee OA.^24^ We used the KOOS subscale values both as continuous values and according to the patient acceptable symptom state (PASS) score as estimated previously in people with ACLR,^16^ giving the following thresholds: pain, 88.9; symptoms, 57.1; ADL, 100; sport/rec, 75; and knee-related QoL, 62.5. Each KOOS subscale was converted to a binary variable based on the PASS threshold.

### Statistical Methods

Descriptive results were given as means and standard deviations, or frequency and percentages. Analyses comparing participants included in study assessments with those not included, were conducted using both parametric and nonparametric tests. Cartilage morphology (dichotomized into no abnormalities and abnormalities [Grade 0 vs Grade ≥1], subsurface cartilage matrix (UTE-T2* values measured in milliseconds), and knee function variables (KOOS subscales) were considered dependent variables. Daily step count was the independent variable. We standardized the steps per day variable to increments of 1 SD (3199 steps per day) for all analyses. The following statistical models were used: (1) binary logistic regression analysis was conducted to analyze the association between daily step count and cartilage morphology status for each area in the femoral condyle and tibia plateau, (2) linear regression analysis was used to assess the association between daily step count and subsurface cartilage matrix UTE-T2* in each medial and lateral femoral and tibial subregion, (3) linear regression was used for analyzing daily step count with all the KOOS subscales (0-100), and (4) binary logistic regression was used to analyze the associations between daily step count and KOOS PASS status for each subscale. All analyses except cartilage morphology were adjusted for the possible confounding factors age, sex, and BMI because these variables might influence both daily step count and development of knee osteoarthritis and knee function. Cartilage morphology had few cases in the response variable; thus, we adjusted for BMI, which has been shown to increase risk for post-traumatic knee OA.^10^ SPSS Version 27 was used for the statistical analyses.

## Results

**Figure 2.**
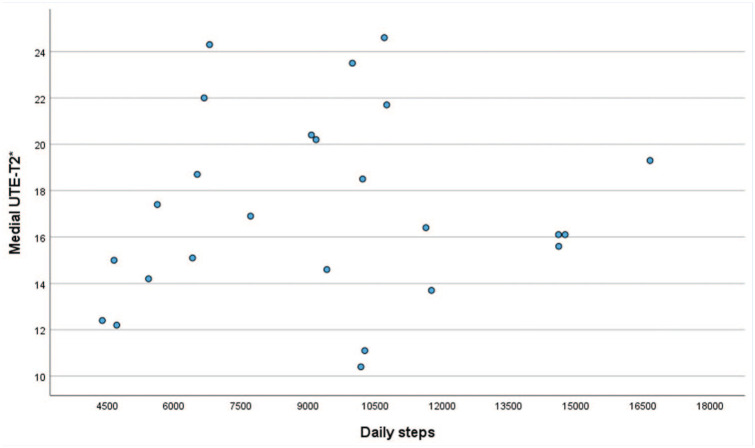
Scatter plot of daily steps and medial UTE-T2* values (milliseconds).

A total of 34 participants (18 females and 16 males) with ACLR were analyzed (68% of the sample of 50). There were no significant differences in mean age (30.6 [7.8] vs 33.4 [10.8] years), mean BMI (24.8 [2.6] vs 24.2 [3.4] kg/m^2^) or KOOS median values (pain, 96 vs 96; symptoms, 91 vs 94; ADL, 94 vs 94; sport/rec, 83 vs 88; QoL, 55 vs 56) between the participants analyzed (n = 34) and those not analyzed (n = 16). Medial meniscal tear at the time of surgery was reported for 6 participants (18%), and lateral tear for 13 (38%) participants. Graft types included 7 Achilles tendon allografts, 7 patellar tendon allografts, 10 patellar tendon autografts, 5 hamstring autografts, 2 peroneus longus allografts, and 1 quadriceps tendon autograft, while 2 had unreported graft types. Mean (SD) daily step counts were 9276 (3199). Descriptive data for cartilage morphology are shown in Table 1. Subsurface cartilage matrix data included 25 of 34 participants with reliable UTE-T2* values. Mean (SD) UTE-T2* values were 17.3 (4.3) milliseconds for MFC and 17.1 (4.6) milliseconds for MTP. For LFC, the mean UTE-T2* value was 16.1 (3.7) milliseconds, with 14.7 (3.5) milliseconds for LTP. Figure 2 depicts the association between daily steps and UTE-T2* values.

**Table 1.** Descriptive data of cartilage morphology (n = 33*^a^* )

Cartilage tissue	Grade 0	Grade 1	Grade 2	Grade 3
Central MFC	29 (88)	2 (6)	1(3)	1(3)
Posterior MFC	25 (76)	8 (24)	0	0
Central LFC	18 (55)	9 (27)	5 (15)	1 (3)
Posterior LFC	25 (76)	5 (15)	3 (9)	0
Central MTP	33 (100)	0 (0)	0	0
Central LTP	29 (88)	4 (12)	0	0
Posterior LTP	25 (76)	7 (21)	1 (3)	0

LFC, lateral femoral condyle; LTP, lateral tibia plateau; MFC, medial femoral condyle; MTP, medial tibial plateau. Data are presented as number (%). Grades are based on the Outerbridge scoring (0-4).

a One participant had missing MRI data. No participants had Grade 4.

Mean (SD) KOOS values were for pain 94 (7); symptoms 92 (8), ADL 91 (9), sport/rec 85 (14), and QoL 56 (22), and the PASS distribution is shown in Table 2.

**Table 2.** PASS for KOOS

	Pain	Symptoms	ADL	Sport/rec	QoL
Above PASS	28 (82%)	34 (100%)	8 (24%)	22 (65%)	14 (41%)
Below PASS	6 (18%)		26 (76%)	12 (35%)	20 (59%)

ADL, activities of daily living; KOOS, Knee Injury and Osteoarthritis Outcome Score; PASS, patient acceptable symptom state; QoL, quality of life; Sport/rec, sport and recreation. Values are number (%).

### Association Between Daily Step Counts With Cartilage Changes and Knee Symptoms and Function

There were no significant associations between daily step count and cartilage morphology (Table 3). Data from 9 of 34 participants UTE-T2* were unusable due to inadvertent receiver gain change between echo image acquisitions precluding accurate T2* curve fit. Data on 25 participants showed no statistically significant associations between daily step count and UTE-T2* values for the MFC (β = 0.09, *P* = 0.92) and MTP (β = 0.20, *P* = 0.84) or for LFC (β = −0.65, *P* = 0.38) and LTP (β = 0.98, *P* = 0.21). No significant associations were found between daily step counts and KOOS PASS status (Table 4) or KOOS scores (symptoms: β = 0.59, *P* = 0.69; pain: β = 0.22, *P* = 0.86; ADL: β = 1.7, *P* = 0.33; sport/rec: β = 1.4, *P* = 0.59; QOL: β = −0.35, *P* = 0.93).

**Table 3.** Association of daily step count and cartilage morphology

Cartilage tissue (0 vs ≥1)	OR (95% CI)
Posterior MFC	0.93 (0.42, 2.09)
Central LFC	1.16 (0.58, 2.31)
Posterior LFC	0.70 (0.30, 1.65)
Posterior LTP	1.58 (0.69, 3.60)

Numbers represent ORs (95% CIs) for cartilage changes with daily step counts standardized to increments of 1 SD (3199 steps). Analyses are adjusted for BMI. Due to too few cases with cartilage changes ≥1, no analyses were done for the central MFC (n = 4), central MTP (n = 0), or central LTP (n = 4). LFC, lateral femoral condyle; LTP, lateral tibia plateau; MFC, medial femoral condyle; OR, odds ratio.

**Table 4.** Association of daily step count and knee symptoms and function

KOOS subscores	ORs (95% CIs)
Pain (PASS cut off 88.9)	1.12 (0.45, 2.83)
ADL (PASS cut off 100)	1.49 (0.66, 3.37)
Sport/rec (PASS cut off 75)	1.11 (0.54, 2.31)
QoL (PASS cut off 62.5)	1.36 (0.67, 2.78)

Numbers represent ORs (95% CIs) for KOOS subscores with daily step counts standardized to increments of 1 SD (3199 steps). No analyses were conducted for symptoms due to too little variation in data. ADL, activities of daily living; KOOS, Knee Injury and Osteoarthritis Outcome Score; PASS, patient acceptable symptom state. QoL, quality of life; Sport/rec, sport and recreation.

## Discussion

This study revealed no associations between daily step count and MRI evaluations of cartilage morphology or composition in participants with stable knees recruited from the community 2 years after ACLR. Moreover, daily step count did not show relationship with patient-reported outcomes, even though substantially fewer than half of participants reached acceptable levels in ADL and QoL. These findings suggest that daily step count measured by a basic activity tracker alone may not be a reliable discriminator of patient outcomes or cartilage degeneration early after ACLR. A different, or more challenging, measure of knee function than step counts may be needed to better characterize knee function recovery in this population because simple step count may not fully capture knee function in relatively young people with ACLR. Furthermore, the low PASS rates for ADL and QoL in participants 2 years after successful ACLR highlight the need for additional research to improve post-ACLR recovery along with efforts to reduce the risk of OA.

The lack of association between cartilage morphology and daily step count may be because most participants still have normal to near normal appearing cartilage 2 years after ACLR, resulting in too little variation in the data to detect associations. Further, morphological MRI may lack sensitivity to discern early signs of OA, as opposed to compositional MRI.^13^ However, compositional cartilage assessment in this work also showed no association with step count. In addition to having few cartilage abnormalities, the participants in our study had high average KOOS pain scores, reflecting low pain levels. Only 6 participants (18%) scored below the PASS value for pain, indicating that few participants had sufficient knee pain to potentially limit step count. Pain limits activity and participant pain status provides additional insight into our findings.

The lack of correlation between activity and OA has been observed in established knee OA. Data from population cohorts at risk of developing OA or with established knee OA, such as the Multicenter Osteoarthritis Study (MOST) and the Osteoarthritis Initiative (OAI) have not detected a relationship between walking or other objectively measured activity levels such as average daily minutes of moderate-to-vigorous physical activity and structural progression over time.^18,23^ In a recent review of physical activity and joint health, Morgan et al^15^ concluded that, although relatively extensive laboratory research exists suggesting that moderate physical activity can promote knee joint health, including animal studies, human studies provide less definitive conclusions. The authors express the need for objective measures of physical activity to ensure measurement of physiological and mechanical impacts on joint health. A review by Xu et al^36^ summarized studies on physical activity level and features of knee OA on MRIs in people without knee OA. The review included 18 studies but found no strong evidence for presence or absence of associations between physical activity and cartilage features of knee OA on MRI scans. However, none of the reviewed studies included participants with ACL injury.

Thus, our study adds to a growing body of evidence showing that walking may be too mild a physical task with which to elicit or measure substantial cartilage compositional changes in the early years after ACLR for this typically young and active population. A recent cross-sectional study also showed no association between daily steps and proteoglycan density in the ACLR knee as measured by T1rho MRI interlimb ratio.^7^ Another study found that a 30-minute walking session did not contribute to immediate medial femoral cartilage thinning in patients 5 years after ACLR,^19^ contrary to findings in healthy control knees. Beyond walking, a prospective study found that activity in general, as assessed by Tegner scores, showed no relationships with cartilage thickening in the central medial femur or thinning in posterior parts of the femur up to 2 years after ACLR.^12^

A high proportion of young people with ACL injury develop knee OA over time,^2^ but little evidence exists on how to prevent this process early after the injury or reconstruction.^14,30,31^ The question of what activity levels those with knee injuries ideally should maintain to optimize a healthy knee joint health over time remains unanswered. Many patients want to return to high-level pivoting sports, whereas others aim to maintain activity in recreational sports involving walking or running. No evidence-based recommendations exist on level of walking related to what is optimal for preserving cartilage health in knee-injured people. The data from our study showing low PASS rates with KOOS ADL and KOOS QoL in participants 2 years after ACLR suggest compromised function and highlight the need to improve our knowledge on how different activities influence cartilage health in those at increased risk for OA.

The PASS analysis indicates suboptimal recovery in a substantial proportion of participants recruited from the community with stable knees. Consistent with a clinically successful ACL, most participants reported acceptable pain states. Thus, it is notably that only 24% of these participants 2 years after ACLR achieved an acceptable symptom state for ADL function. KOOS ADL involves walking, stair climbing, sit-to-stand, standing still, reaching for items on the floor, entering and exiting a vehicle, and other ADL. Normative value for KOOS ADL in healthy young adults is a score of 100,^20^ indicating that >75% of our study participants reported some impairment with these relatively simple activities. Most patients with ACL injury report improved knee function 1 to 2 years after the injury, but a previous study showed that 1 in 10 patients did not reach optimal knee function.^21^ The PASS criteria may be a useful indicator of poor recovery in ACLR patients at the time when we expect optimal recovery because it gives information on subgroups with poorer knee function, as seen in our study.

### Limitations of the Study

The strengths of this study include the use of sensitive MRI techniques with the possibility to detect early subsurface cartilage matrix changes and objectively measured daily step counts, which is the recommended method to measure physical activity in this population.^6,31^ However, the current study used cross-sectional data, and daily step count and/or UTE-T2* data were not collected for several participants. Thus, only associations on a selected sample could be analyzed, although the subjects analyzed did not seem to differ in age, BMI, or patient-reported outcomes from those who did not have daily step data. In addition, step count does not capture the intensity of activity, and the basic activity tracker used may not have accurately captured all physical activities. Feehan et al^11^ systematically reviewed 67 studies that examined the measurement accuracy of Fitbit activity trackers in a controlled and a free-living setting, and concluded that Fitbit consistently met acceptable accuracy for step count half the time compared with a reference-standard criterion of direct observation and counting of steps in a controlled setting, but with a tendency to underestimate in a controlled setting and overestimate in a free-living setting. However, given that all subjects were assessed with identical Fitbit trackers, any systematic error in the Fitbit estimation of step count would not greatly affect intersubject comparisons. Furthermore, subjects were assessed over a period of multiple days, which would not be feasible for more accurate methods like direct observation.

Given that ACL injury typically occurs in subjects participating in high impact pivoting sports, this study was limited by lack of data on preinjury sport or activity level up to the time of testing at 2 years after ACLR. Few of the study participants had cartilage abnormalities detectable by morphologic MRI or UTE-T2* assessments 2 years after ACLR. Despite evidence for suboptimal recovery in a substantial portion of participants 2 years after ACLR, no significant associations were found between daily step count and cartilage health or knee symptoms and function. Thus, additional work is needed to determine whether more comprehensive measurement of higher demand activities beyond step count measured by a basic activity tracker might detect relationships between activity and cartilage health. Because patient-reported outcomes are more broadly accessible to the community than MRI scans, and as only scant evidence for significant cartilage damage was detected in this cohort anyway, KOOS PASS can provide useful assessment of meaningful functional recovery in ACLR patients. Notably, the low PASS rates for KOOS ADL and KOOS QoL in participants recruited from the community with stable knees after ACLR indicate a need for development and implementation of strategies to optimize treatment and rehabilitation protocols that can improve ACLR recovery.
